# Relationship Between Anxiety Levels, Care Dependency, and Quality of Recovery in Patients Undergoing Orthopedic Surgery

**DOI:** 10.3390/healthcare14162481

**Published:** 2026-08-11

**Authors:** Durdane Yılmaz Güven, Büşra Salar

**Affiliations:** 1 Nursing Department, Faculty of Health Sciences, Karabuk University, Karabuk 78050, Türkiye; 2 Nursing Department, Graduate Education Institute, Karabuk University, Karabuk 78050, Türkiye; busraslr71@gmail.com

**Keywords:** orthopedic surgery, anxiety, care dependency, quality of recovery, postoperative period, nursing care

## Abstract

**Background and Objectives**: Postoperative recovery after orthopedic surgery is associated with psychological and functional factors, yet the independent associations of anxiety and care dependency with postoperative recovery remain unclear. This study aimed to identify factors independently associated with postoperative quality of recovery and to examine the relationships among anxiety, care dependency, and recovery quality. **Methods**: This analytical cross-sectional study included 155 patients who underwent orthopedic surgery. Data were collected on the first postoperative day using a Patient Identification Form, the Beck Anxiety Inventory, the Care Dependency Scale, and the Quality of Recovery-40 (QoR-40) questionnaire through face-to-face interviews. Data were analyzed using descriptive statistics, Mann–Whitney U, Kruskal–Wallis, Spearman’s rank correlation, and multivariable linear regression analyses. **Results**: Older age, female sex, and hypertension and/or diabetes mellitus were significantly associated with higher anxiety and greater care dependency (*p* < 0.05), whereas older age was significantly associated with poorer quality of recovery (*p* < 0.05). Anxiety was negatively correlated with quality of recovery (r = −0.707, *p* < 0.001) and functional independence (r = −0.367, *p* < 0.001), whereas functional independence was positively correlated with quality of recovery (r = 0.557, *p* < 0.001). Multivariable linear regression analysis showed that higher anxiety (β = −0.52), greater functional independence (β = 0.31), and older age (β = −0.18) were independently associated with quality of recovery. **Conclusions**: Older age, female sex, and hypertension and/or diabetes mellitus were independently associated with higher anxiety and greater care dependency. Higher anxiety and lower functional independence were independently associated with poorer postoperative recovery. These findings support the integration of psychological assessment and functional evaluation into routine postoperative nursing care.

## 1. Introduction

Musculoskeletal disorders are among the leading causes of disability worldwide and are associated with substantial physical, psychological, and functional impairments that reduce quality of life and functional independence [1,2]. Although their prevalence increases with age, orthopedic disorders occur across all adult age groups and frequently require surgical intervention [1,2,3,4]. Patients undergoing orthopedic surgery commonly experience postoperative pain, reduced muscle strength, limitations in activities of daily living, anxiety, and temporary loss of functional independence, all of which have been associated with poorer postoperative recovery [3,4,5,6]. Recent evidence has shown that psychological factors, particularly emotional distress, are independently associated with poorer postoperative recovery outcomes in orthopedic patients [5]. Postoperative anxiety, characterized by fear, uncertainty, and apprehension, has been associated with poorer physical recovery, emotional well-being, and participation in postoperative care. Furthermore, reduced functional independence due to disability or activity intolerance may delay patients’ return to their previous roles and daily activities [3,4,5,6].

Assessing patients’ level of independence and dependence provides nurses with essential information for planning individualized nursing care. Care dependency describes the degree to which individuals require support because of reduced self-care abilities [7,8]. Evaluating care dependency enables nurses to identify patients requiring additional support and implement appropriate nursing interventions, thereby facilitating individualized care planning [7,8].

The ability to resume normal activities after surgery and anesthesia is considered an important indicator of successful postoperative recovery [9]. Quality of recovery is a multidimensional concept encompassing physical comfort, emotional well-being, physical independence, pain control, and overall recovery following surgery [6]. Optimizing these dimensions represents one of the primary goals of postoperative nursing care.

Previous studies have shown that psychological factors are associated with postoperative recovery in orthopedic patients. For example, Khalil et al. (2025) reported that emotional distress was independently associated with poorer postoperative recovery following orthopedic surgery [5], whereas Gumus et al. (2026) reported that preoperative surgical fear was independently associated with poorer quality of recovery after total joint arthroplasty [3]. Similarly, Nacakçıoğlu and Açıl (2025) [10] highlighted the importance of care dependency in postoperative recovery following total knee arthroplasty. However, these studies have investigated psychological and functional factors separately [3,5,10]. Evidence simultaneously evaluating postoperative anxiety, care dependency, and quality of recovery while accounting for relevant sociodemographic and clinical characteristics remains limited. Understanding these interrelationships may facilitate the identification of patients at greater risk of poor postoperative recovery and support the development of individualized nursing interventions targeting both psychological and functional needs.

Therefore, this study aimed to examine the associations between selected sociodemographic and clinical characteristics and postoperative outcomes, as well as the relationships among anxiety, care dependency, and quality of recovery in patients undergoing orthopedic surgery.

Research Questions:

1. Are selected sociodemographic and clinical characteristics associated with postoperative anxiety, care dependency, and quality of recovery among patients undergoing orthopedic surgery?
2. What are the relationships among postoperative anxiety, care dependency, and quality of recovery in patients undergoing orthopedic surgery?

## 2. Materials and Methods

This descriptive and correlational study was conducted between April and June 2024 in the Orthopedics and Traumatology inpatient ward of a tertiary training and research hospital in Türkiye. The ward provides preoperative and postoperative inpatient care for adults undergoing elective and emergency orthopedic surgical procedures. During the study period, all patients who met the eligibility criteria were consecutively invited to participate. Because this was a hospital-based cross-sectional study, the final sample consisted of all eligible patients available during the predefined data collection period. The study sample consisted of patients aged 18 years or older who had undergone primary orthopedic surgery, were hospitalized in the Orthopedics and Traumatology inpatient ward, were able to read and communicate in Turkish, and had no cognitive, emotional, or verbal communication problems that could interfere with completion of the study questionnaires. Patients undergoing revision surgery were excluded because their postoperative recovery trajectory differs substantially from that of primary orthopedic procedures, which could have confounded the study outcomes. Patients admitted to the intensive care unit and those who declined to participate were also excluded from the study.

### 2.1. Data-Collection Tools

Demographic and Clinical Characteristics Form: This form, prepared in line with the literature, consists of nine questions, including six items related to sociodemographic characteristics (age, sex, education level, marital status, place of residence, living environment, and employment status) and three items related to health status (presence of chronic disease, previous surgical intervention, and type of anesthesia) [2,6].

BECK Anxiety Scale: The Beck Anxiety Inventory (BAI), developed by Beck et al. in 1988 [11], is a self-report instrument consisting of 21 items that assesses the severity of anxiety symptoms. Each item is scored on a 4-point Likert scale (0–3), yielding a total score ranging from 0 to 63, with higher scores indicating greater anxiety severity. Total scores of 0–7, 8–15, 16–25, and 26–63 indicate minimal, mild, moderate, and severe anxiety, respectively. The Turkish validity and reliability study of the BAI was conducted by Ulusoy et al. [11], who reported a Cronbach’s alpha coefficient of 0.88. In the present study, the median BAI score was 8 (Q1–Q3: 4–17), and the Cronbach’s alpha coefficient was 0.902.

Care Dependency Scale: The Care Dependency Scale (CDS), developed by Dijkstra in the Netherlands in 1999 [12], is a 17-item, single-dimensional instrument that assesses patients’ level of care dependency in activities of daily living. Each item is rated on a 5-point Likert scale ranging from 1 (completely dependent) to 5 (almost/completely independent). Total scores range from 17 to 85, with higher scores indicating greater independence and lower scores indicating greater care dependency. The Turkish validity and reliability study of the CDS was conducted by Yönt et al. in 2010 [12,13], who reported a Cronbach’s alpha coefficient of 0.91. In the present study, the median CDS score was 68 (Q1–Q3: 54–74), and the Cronbach’s alpha coefficient was 0.945.

Quality of Recovery-40 questionnaire [QoR-40]: The Quality of Recovery-40 questionnaire (QoR-40) was developed by Myles et al. in 2000 to assess patients’ quality of recovery following anesthesia and surgery [14]. The questionnaire consists of 40 items grouped into five subscales: emotional state (9 items), physical comfort (12 items), psychological support (7 items), physical independence (5 items), and pain (7 items). Each item is rated on a 5-point Likert scale, yielding a total score ranging from 40 to 200, with higher scores indicating better quality of recovery. The Turkish validity and reliability study was conducted by Karaman et al. in 2014 [15], who reported a Cronbach’s alpha coefficient of 0.94. In the present study, the median QoR-40 score was 175 (Q1–Q3: 158–185), and the Cronbach’s alpha coefficient was 0.96.

### 2.2. The Implementation of the Study

On the first postoperative day, eligible patients who were clinically stable and able to communicate effectively were informed about the purpose of the study, and written informed consent was obtained from those who agreed to participate. The questionnaires were then administered by the researcher through face-to-face interviews. The first postoperative day was selected because patients had recovered from the immediate effects of anesthesia and were clinically able to respond to the questionnaires, while anxiety, care dependency, and quality of recovery could still be assessed during the early postoperative period before hospital discharge. Completion of the questionnaires required approximately 20 min for each participant.

### 2.3. Statistical Analysis

Data were analyzed using IBM SPSS Statistics version 27.0 (IBM Corp., Armonk, NY, USA). Descriptive statistics were presented as frequencies and percentages for categorical variables and as medians with interquartile ranges (Q1–Q3) for continuous variables. The normality of the Beck Anxiety Inventory, Care Dependency Scale, and Quality of Recovery-40 scores was assessed using the Kolmogorov–Smirnov test. As the data did not meet the assumption of normality, non-parametric statistical tests were applied. The Mann–Whitney U test was used for comparisons between two independent groups, and the Kruskal–Wallis test was used for comparisons among three or more independent groups. Associations between anxiety, care dependency, and quality of recovery scores were examined using Spearman’s rank correlation analysis. Multivariable linear regression analyses were performed to examine factors independently associated with anxiety, care dependency, and quality of recovery. Prior to the regression analyses, model assumptions were evaluated. Multicollinearity was assessed using variance inflation factors (VIFs), with all values below 1.3. The independence of residuals was assessed using the Durbin–Watson statistic (1.86–2.08). Normality of residuals and homoscedasticity were evaluated using normal probability plots, histograms, and residual scatterplots, and all regression assumptions were considered acceptable. Regression results are presented as standardized regression coefficients (β), 95% confidence intervals (CIs), *p*-values, and coefficients of determination (R^2^). A two-sided *p* value < 0.05 was considered statistically significant.

### 2.4. Ethical Approval

Prior to commencing the study, permission was obtained from the Karabuk Non-Interventional Research Ethics Committee (Date: 11 February 2024, Number: E-77192459-050.99-319580 Decision No 2024/1653). Institutional permission was obtained from the Karabuk Training and Research Hospital where the study was conducted (date: 27 March 2024, Number: E-34771223-774.99-240252912). This study was conducted in accordance with the principles of the Declaration of Helsinki.

## 3. Results

### 3.1. Descriptive Characteristics

Among the patients included in the study, 61.3% were older adults, 60.6% were female, 52.3% had completed primary education, 85.8% were married, 43.9% lived in provincial centers, and 60.0% were unemployed. In addition, 61.9% had a chronic disease (hypertension and/or diabetes mellitus), 37.4% underwent total knee arthroplasty (TKA), and 77.4% received regional anesthesia. The median Beck Anxiety Inventory score was 8 (Q1–Q3: 4–17), the median Care Dependency Scale score was 68 (Q1–Q3: 54–74), and the median total Quality of Recovery-40 score was 175 (Q1–Q3: 158–185). The corresponding median subscale scores were 2 (Q1–Q3: 1–4) for Physical independence, 52 (Q1–Q3: 45–57) for Physical Comfort, 41 (Q1–Q3: 35–44) for Emotional state, 1 (Q1–Q3: 0–4) for Patient Support, and 30 (Q1–Q3: 28–33) for Pain (Table 1).

### 3.2. Comparisons by Sociodemographic and Clinical Characteristics

As shown in Table 2, older age, female sex, and the presence of a chronic disease were significantly associated with higher anxiety levels (*p* < 0.05). Similarly, older age, female sex, and the presence of a chronic disease were significantly associated with greater care dependency (*p* < 0.05). Regarding quality of recovery, older age was significantly associated with lower overall recovery quality and lower comfort subscale scores (*p* < 0.05) (Table 2). Although surgical procedure was significantly associated with care dependency in the univariate analysis (Table 2), this association was no longer statistically significant after adjustment for age, sex, and chronic disease in the multivariable regression model, suggesting that the univariate association may have been confounded by these covariates.

### 3.3. Correlations Among Anxiety, Care Dependency, and Quality of Recovery

A weak negative correlation was found between the Beck Anxiety Inventory and the Care Dependency Scale, whereas moderate negative correlations were observed between the Beck Anxiety Inventory and the Quality of Recovery-40 total and subscale scores (*p* < 0.001). In addition, moderate positive correlations were observed between the Care Dependency Scale and the Quality of Recovery-40 total and subscale scores (*p* < 0.001) (Table 3).

### 3.4. Factors Independently Associated with Anxiety, Care Dependency, and Quality of Recovery

Multivariable linear regression analyses were performed to identify factors independently associated with anxiety, care dependency, and quality of recovery among patients undergoing orthopedic surgery. Sociodemographic and clinical variables, including age, sex, presence of chronic disease, and surgical procedure, were entered into the regression models. Older age, female sex, and the presence of chronic disease were independently associated with higher anxiety scores, whereas surgical procedure was not significantly associated with anxiety. Similarly, older age, female sex, and the presence of chronic disease were independently associated with greater care dependency. For quality of recovery, higher anxiety scores and older age were independently associated with poorer recovery, whereas greater independence (higher Care Dependency Scale scores) was independently associated with better quality of recovery. The final regression models explained 27%, 31%, and 46% of the variance in anxiety, care dependency, and quality of recovery scores, respectively (Table 4).

## 4. Discussion

The present study investigated the relationships among anxiety, care dependency, and quality of recovery in patients undergoing orthopedic surgery while identifying factors independently associated with these outcomes. Multivariable regression analyses showed that older age, female sex, and the presence of chronic disease were independently associated with higher anxiety levels and greater care dependency. Furthermore, older age and higher anxiety scores were independently associated with poorer quality of recovery, whereas greater functional independence, reflected by higher Care Dependency Scale scores, was independently associated with better quality of recovery. These findings suggest that postoperative recovery is closely associated with not only surgical factors but also psychological and functional characteristics, emphasizing the importance of comprehensive nursing assessment and individualized postoperative care.

### 4.1. Age

Population ageing has led to a growing number of older adults requiring orthopedic surgical interventions, making the maintenance of functional independence and postoperative recovery an important healthcare priority [1,4,5]. Consistent with previous studies, more than half of the participants in the present study were older adults. More importantly, regression analyses showed that older age was independently associated with higher anxiety, greater care dependency, and poorer quality of recovery. These findings suggest that older age remains an important factor associated with postoperative outcomes, even after controlling for other patient characteristics.

Previous studies have shown that older adults are at greater risk of postoperative anxiety, which has been associated with frailty, comorbidities, reduced mobility, and other age-related factors [4,16,17]. Similarly, recovery following orthopedic surgery is often slower in older adults because frailty, chronic diseases, polypharmacy, malnutrition, and cognitive decline reduce physiological reserve and functional capacity [16,18]. Moreover, older patients frequently require prolonged rehabilitation, particularly following hip fracture surgery, and age-related functional decline has consistently been associated with increased care dependency [19,20,21]. The findings of the present study are consistent with this evidence and further suggest that older age is independently associated with psychological distress, reduced functional independence, and poorer postoperative recovery. These results highlight the importance of comprehensive geriatric assessment, early identification of vulnerable patients, and individualized multidisciplinary nursing interventions that address both physical and psychological needs throughout the perioperative period [4,16,22].

### 4.2. Chronic Disease and Surgical Procedure

Multivariable regression analysis showed that the presence of chronic disease was independently associated with both higher anxiety levels and greater care dependency. This finding is consistent with previous studies showing that individuals with chronic diseases experience higher levels of anxiety, greater functional limitations, and increased care needs, all of which are associated with poorer postoperative recovery [20,23]. Likewise, longitudinal studies conducted in older orthopedic patients have identified comorbidity as one of the principal determinants of care dependency following surgery [16,20]. In the present study, patients with hypertension and/or diabetes mellitus had higher anxiety scores and greater care dependency than those without chronic disease, suggesting that chronic conditions may be associated with impaired functional capacity, delayed recovery, and an increased need for nursing support. These findings emphasize the importance of comprehensive preoperative assessment and individualized postoperative care for patients with chronic diseases [16].

Although care dependency differed according to surgical procedure, surgical procedure was not independently associated with anxiety or care dependency in the multivariable regression analysis. This finding suggests that patient-related characteristics, particularly age, sex, and chronic disease, may be more closely associated with postoperative outcomes than the type of orthopedic procedure itself. Nevertheless, patients undergoing total hip arthroplasty had higher care dependency and reduced mobility during the early postoperative period, which is consistent with previous studies reporting that older patients undergoing hip surgery often experience greater functional impairment, increased care dependency, and reduced functional capacity during postoperative recovery [20,21,24]. The absence of significant differences in anxiety and quality of recovery between surgical procedures may reflect the fact that orthopedic procedures share common postoperative challenges, including pain, mobility limitations, and functional impairment, resulting in broadly comparable recovery experiences across different surgical procedures [3,5].

### 4.3. Relationship Among Anxiety, Care Dependency, and Quality of Recovery

One of the principal findings of the present study was the close relationship among anxiety, care dependency, and quality of recovery in patients undergoing orthopedic surgery. Correlation analyses showed that higher anxiety was associated with greater care dependency and poorer quality of recovery, whereas greater functional independence, reflected by higher Care Dependency Scale scores, was associated with better quality of recovery. Furthermore, multivariable regression analysis showed that higher anxiety was independently associated with poorer quality of recovery, whereas greater functional independence was independently associated with better quality of recovery. These findings suggest that psychological well-being and functional status are closely interconnected during the postoperative period and should be considered together when planning individualized nursing care [5,10,22].

The relationship between anxiety and care dependency may be explained by the reciprocal interaction between psychological distress and functional performance. Following orthopedic surgery, patients frequently experience pain, mobility limitations, fear of movement, and uncertainty regarding their recovery, all of which have been associated with poorer psychological well-being and functional recovery [3,5,25]. These factors may reduce patients’ willingness to participate in self-care activities and increase their dependence on caregivers [20,26]. Conversely, greater care dependency may contribute to feelings of helplessness, loss of autonomy, and psychological distress, further compromising postoperative recovery [5,26]. Previous studies have similarly reported that psychological factors and functional status are closely associated with care dependency and postoperative recovery among orthopedic patients [5,20,26]. Collectively, these findings indicate that anxiety and care dependency should not be considered isolated outcomes but rather closely interrelated components of postoperative recovery [5,10].

The present study also showed that higher anxiety was independently associated with poorer quality of recovery, whereas greater functional independence was independently associated with better quality of recovery. This finding is consistent with previous studies reporting that anxiety and psychological distress are associated with poorer postoperative recovery, greater pain perception, and reduced recovery outcomes among orthopedic patients [3,5,17,22,27]. Likewise, previous studies have consistently shown that preserving functional independence through early mobilization, rehabilitation, and continuous nursing care is associated with improved functional outcomes, enhanced quality of life, and better postoperative recovery among orthopedic patients [24,28]. Taken together, these findings suggest that postoperative recovery is closely associated with not only surgical factors but also patients’ psychological adaptation and functional capacity. Therefore, routine assessment of anxiety and functional independence, together with individualized nursing interventions aimed at reducing psychological distress and promoting self-care, may contribute to improved postoperative recovery outcomes [22,28,29].

These findings underscore the importance of integrating psychological assessment with the evaluation of functional independence throughout the postoperative period [5,10]. Nurses are well positioned to identify patients at increased risk of anxiety and functional dependence, implement individualized interventions, and coordinate multidisciplinary care to support recovery and promote functional independence [4,16,22,28].

## 5. Limitations

This study has several limitations. First, it was conducted at a single tertiary training and research hospital, which may limit the generalizability of the findings. Second, the cross-sectional design precludes any inference of causal relationships among anxiety, care dependency, and quality of recovery. Third, data were collected only during the early postoperative period; therefore, the findings reflect early postoperative recovery rather than long-term recovery outcomes.

## 6. Conclusions

This study found that postoperative anxiety, care dependency, and quality of recovery were closely interrelated in patients undergoing orthopedic surgery. Multivariable regression analysis showed that older age, female sex, and the presence of chronic disease were independently associated with higher anxiety levels and greater care dependency. In addition, older age and higher anxiety were independently associated with poorer quality of recovery, whereas greater functional independence, reflected by higher Care Dependency Scale scores, was independently associated with better quality of recovery.

These findings highlight the importance of integrating psychological assessment and the evaluation of functional independence into routine postoperative nursing care. Early identification of patients at increased risk of anxiety and care dependency may facilitate individualized nursing interventions, promote functional independence, and improve recovery outcomes. Particular attention should be given to older adults and patients with chronic diseases, who may require more comprehensive postoperative support.

Future longitudinal and randomized controlled studies are needed to clarify the causal relationships among anxiety, care dependency, and quality of recovery and to evaluate the effectiveness of nursing interventions aimed at reducing anxiety, enhancing functional independence, and improving postoperative recovery.

## Figures and Tables

**Table 1:** Descriptive Characteristics of Patients, Health Status Findings, and Median (Q1–Q3) Scale Scores.

			*n*	%
Age		Younger adults (<65 years)	60	38.7
		Older adults (≥65 years)	95	61.3
Gender		Woman	94	60.6
		Male	61	39.4
Education level		Illiterate	37	23.9
		Primary education	81	52.3
		High school and above	37	23.9
Marital status		Single	22	14.2
		Married	133	85.8
Place of residence		Village	31	20.0
		District/town	56	36.1
		Provincial center	68	43.9
Employment status		Unemployed	93	60.0
		Employed	62	40.0
Presence of chronic disease		Hypertension and/or diabetes mellitus	96	61.9
		None	59	38.1
Surgical procedure		Arthrosis	47	30.3
		TKA	50	32.3
		THA	58	37.4
Type of anesthesia		General anesthesia	35	22.6
		Regional anesthesia	120	77.4
			Median	Q1–Q3
Beck Anxiety Scale			8	4–17
Cronbach’s Alpha			0.902	
Care Dependence Scale			68	54–74
Cronbach’s Alpha			0.945	
Quality of Recovery-40 (QoR-40)	Physical independence		2	1–4
	Cronbach’s Alpha		0.823	
	Physical comfort		52	45–57
	Cronbach’s Alpha		0.913	
	Emotional state		41	35–44
	Cronbach’s Alpha		0.885	
	Patient Support		1	0–4
	Cronbach’s Alpha		0.937	
	Pain		30	28–33
	Cronbach’s Alpha		0.827	
	Total		175	158–185
	Cronbach’s Alpha		0.96	

**Table 2:** Comparison of Beck Anxiety Inventory, Care Dependency Scale, and Quality of Recovery-40 Scores According to Selected Patient Characteristics.

	Beck Anxiety Scale	Care Dependency Scale	Quality of Recovery-40 (QoR-40)					
			Physical Independence	Physical Comfort	Emotional State	Patient Support	Pain	Total
	Median (Q1–Q3)	Median (Q1–Q3)	Median (Q1–Q3)	Median (Q1–Q3)	Median (Q1–Q3)	Median (Q1–Q3)	Median (Q1–Q3)	Median (Q1–Q3)
Age								
Younger adults (<65 years)	4 (3–10.9)	71 (61.75–75)	1 (0–2)	53.5 (46–58.25)	42 (37.75–44.25)	1 (0–2)	31 (28–33)	180 (165.75–190.25)
Older adults (≥65 years)	9 (5–19)	63 (52–72)	2 (1–4)	51 (45–56)	39 (34.5–43)	2 (0–5)	30 (27–33)	167 (155–181)
Z	3.137	−2.509	−0.649	−3.299	−2.003	−1.710	−1.525	−2.923
*p*	0.002	0.012	0.516	<0.001	0.045	0.087	0.127	0.003
Chronic disease								
Yes	5 (3–13.5)	72 (63–75.5)	2 (0–2.5)	54 (47.5–59)	42 (36–44)	1 (0–2)	31 (28–33)	180 (160–189.5)
None	9 (4–18.25)	62 (51–71)	2 (1–4)	51 (44.75–56)	39 (35–43.25)	2 (0–5)	30 (27–33)	169 (156.75–183)
Z	2480	−3.818	3.810	6.593	0.063	1.573	1.008	−1.952
*p*	0.013	<0.001	0.980	0.010	0.802	0.210	0.315	0.051
Surgical procedure								
Arthrosis	8 (3–19)	72 (63–76.5)	1 (1–4)	53 (46.5–57.5)	4 (34.5–44)	1 (0–3)	31 (28–33)	180 (161.5–191)
TKA	9 (4.25–18.75)	61.5 (51–71)	2 (1–4)	50 (55.75)	39 (36–44)	1.5 (0.25–5.75)	30 (28–32.5)	166.5 (155.5–182.75)
THA	6 (4–13)	67 (58–74)	2 (1–3)	52 (44–57)	41 (35–43)	2 (0–3)	31 (28–33)	174 (159–185)
χ^2^	3.489	10.348	13.291	0.737	2279	1.666	0.421	3.636
*p*	0.175	0.016	0.001	0.692	0.320	0.435	0.810	0.162
Difference	-	2 < 1 = 3	2 = 3 < 1	-	-	-	-	-

**Table 3:** Relationships Between the Beck Anxiety Inventory, Care Dependency Scale, and Quality of Recovery-40 (QoR-40).

		1	2	3	4	5	6	7	8
1. Beck Anxiety Scale	r								
	*p*								
2. Care Dependency Scale	r	−0.367							
	*p*	<0.001							
3. Quality of Recovery (QoR-40)	r	−0.707	0.557						
	*p*	<0.001	<0.001						
4. Physical independence	r	−0.401	0.680	0.750					
	*p*	<0.001	<0.001	<0.001					
5. Physical comfort	r	−0.676	0.422	0.883	0.538				
	*p*	<0.001	<0.001	<0.001	<0.001				
6. Emotional state	r	−0.691	0.424	0.883	0.540	0.752			
	*p*	<0.001	<0.001	<0.001	<0.001	<0.001			
7. Patient Support	r	−0.410	0.484	0.674	0.516	0.476	0.557		
	*p*	<0.001	<0.001	<0.001	<0.001	<0.001	<0.001		
8. Pain	r	−0.547	0.447	0.707	0.377	0.561	0.599	0.460	
	*p*	<0.001	<0.001	<0.001	<0.001	<0.001	<0.001	<0.001	

**Table 4:** Multivariable Linear Regression Models Examining Factors Independently Associated with Anxiety, Care Dependency, and Quality of Recovery.

Outcome Variable	Independent Variable	Standardized β	95% CI	*p*-Value
Beck Anxiety Inventory	Age	0.21	0.08 to 0.34	0.003
	Female sex	0.18	0.04 to 0.31	0.012
	Chronic disease	0.19	0.05 to 0.33	0.008
	Surgical procedure	NS	—	>0.05
	Model fit	R^2^ = 0.27; Adj. R^2^ = 0.25; F = 18.62; *p* < 0.001		
Care Dependency Scale	Age	−0.24	−0.37 to −0.11	0.001
	Female sex	−0.12	−0.24 to −0.01	0.048
	Chronic disease	−0.22	−0.36 to −0.08	0.004
	Surgical procedure	NS	—	>0.05
	Model fit	R^2^ = 0.31; Adj. R^2^ = 0.29; F = 22.71; *p* < 0.001		
Quality of Recovery (QoR-40)	Anxiety score	−0.52	−0.63 to −0.41	<0.001
	Care dependency	0.31	0.19 to 0.43	<0.001
	Age	−0.18	−0.33 to −0.03	0.015
	Model fit	R^2^ = 0.46; Adj. R^2^ = 0.45; F = 42.85; *p* < 0.001		

Abbreviations: β = standardized regression coefficient; CI = confidence interval; NS = not significant.

## Data Availability

The data are not publicly available due to privacy restrictions.

## Abbreviation

QoR-40	Quality of Recovery-40 questionnaire
